# Blood pressure management after endovascular thrombectomy: Insights of recent randomized controlled trials

**DOI:** 10.1111/cns.14907

**Published:** 2024-08-08

**Authors:** Xiao Dong, Yuanyuan Liu, Xuehong Chu, Erlan Yu, Xiaole Jia, Xunming Ji, Chuanjie Wu

**Affiliations:** ^1^ Department of Neurology, Xuanwu Hospital Capital Medical University Beijing China; ^2^ Department of Neurosurgery, Xuanwu Hospital Capital Medical University Beijing China

**Keywords:** acute ischemic stroke, blood pressure, endovascular thrombectomy, randomized controlled trials

## Abstract

**Background:**

The ideal blood pressure (BP) target in patients who undergo endovascular thrombectomy (EVT) with successful reperfusion is uncertain. Observational studies show that elevated BP during this period is associated with a higher risk of intracranial hemorrhage (ICH) and worse clinical outcomes. Several randomized controlled trials (RCTs) have explored whether intensive BP lowering improves clinical outcomes in these patients.

**Aims:**

This review aims to summarize the recent RCTs that compare intensive and conventional BP management strategies following EVT and discuss the innovative directions to improve.

**Result:**

The recently published RCTs failed to demonstrate the benefit of intensive BP control on the functional outcome and decreasing the risk of ICH. The complex mechanism in cerebral blood flow regulation and the inappropriate BP range chosen in RCTs may be the reasons behind the inconsistent results between observational studies and RCTs. Individualized BP management, reducing BP variability, and multi‐stage BP management should be paid more attention in future exploration.

**Conclusion:**

Intensive BP target did not improve clinical outcomes after successful EVT as compared with a conventional BP target. Further research is required to identify the optimal BP management strategy after reperfusion.

## Graphical abstract

The ideal systolic blood pressure target following successful endovascular thrombectomy remains unclear. This article summarizes and analyzes the key conclusions and implications of recent randomized controlled trials, while also exploring the innovative directions for optimizing blood pressure control following endovascular thrombectomy. The recently completed randomized controlled trials failed to demonstrate the beneficial effect of intensive blood pressure control on both the functional outcome and reduction of the risk of intraparenchymal hemorrhage. Future research should place greater emphasis on individualized blood pressure management strategies, minimizing blood pressure variability, and implementing multi‐stage BP management protocols in this field.

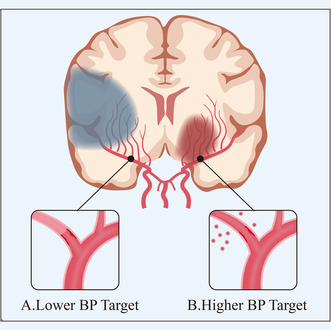

## 1 INTRODUCTION

After endovascular thrombectomy (EVT), more than 50% of the patients did not gain functional independence as expected, even when the images demonstrated clearly that the arterial occlusion had been recanalized.^1^, ^2^, ^3^ Adjunctive therapies are still necessary to improve outcomes in patients suffering from large vessel occlusion (LVO) stroke. Recently, evidence shows that higher blood pressure (BP) is associated with undesirable outcomes and a higher risk of intracranial hemorrhage (ICH).^4^, ^5^, ^6^, ^7^ Lower BP, on the other hand, may result in less cerebral perfusion, which could exacerbate ischemia and infarction. The appropriate SBP value following successful reperfusion is controversial. Consequently, optimizing the management of systolic blood pressure (SBP) in these patients may be a useful therapeutic strategy to lessen unfavorable outcomes. Nowadays, as recommended by guidelines of the American Stroke Association (ASA) and European Stroke Organization (ESO), BP should be targeted in the range of <180/105 mmHg within the first 24 h after successful EVT.^8^, ^9^ However, there is not high‐quality evidence to back up this threshold. Therefore, the ideal BP target is still unclear. In addition, a more aggressive BP treatment is frequently used in the clinical practice of American hospitals.^10^ There is an immediate need for more persuasive data to back up more persuasive BP control measures.

Which is the best method of SBP lowering: more intensive, intensive, moderate, or conventional? Several randomized controlled trials (RCTs) and a large number of cohort studies have provided a variety of evidence for this topic. Although the clinical studies are heterogeneous in terms of approaches to lowering BP, BP target grouping, and definition in observational studies, meta‐analyses, and reviews, the general findings showed a trend that lower SBP is associated with a better prognosis and less cerebral hemorrhage. A prospective observational analysis found that a peak SBP of around 160 mm Hg in the first 24 h after EVT separated individuals with favorable vs. poor functional results. Another study demonstrated a positive association between improved patient outcomes and moderate SBP readings between 140 and 160 mm Hg.^4^, ^5^, ^6^, ^11^, ^12^, ^13^, ^14^ However, the causality is uncertain due to the methodological limitations of these studies since complex underlying confounders may not be eliminated. Based on the evidence of observational studies, several RCTs with varied groupings of BP target levels have been completed or are in progress. We summarize them as follows.

## 2 RECENT RCTs EVIDENCE

At present, there are eight registered randomized controlled studies conducted, including the Blood Pressure Target in Acute Stroke to Reduce hemorrhage After Endovascular Therapy (BP‐TARGET) trial,^15^ Second Enhanced Control of Hypertension and Thrombectomy Stroke Study (ENCHANTED2/MT) trial,^16^ Patients Treated With Intra‐arterial Thrombectomy Optimal Blood Pressure Control (OPTIMAL‐BP) trial,^17^ Blood Pressure After Endovascular Stroke Therapy‐II (BEST‐II) trial,^18^ and the three ongoing trials, Intensive Control of Blood Pressure in Acute Ischemic Stroke (CRISIS I) trial (NCT04775147), Blood Pressure Management in Stroke Following Endovascular Treatment (DETECT) trial (NCT04484350), Clevidipine Infusion for Blood Pressure Management After Successful Revascularization in Acute Ischemic Stroke (CLEVER) trial (NCT05175547), and Hemodynamic Optimization of Cerebral Perfusion After Endovascular Therapy in Patient With Acute Ischemic Stroke (HOPE) trial (NCT04892511). The comprehensive findings of these trials could shed light on optimal BP regulation after EVT for acute ischemic stroke. In this article, we describe the most recent developments in randomized controlled studies chronologically to introduce the status quo and provide inspiration for future work.

Over the past 3 years, a series of high‐quality evidence has been produced by randomized controlled studies (Table 1). Unlike the subsequent trials, which evaluated the effects on functional outcome at 90 days, the BP‐TARGET trial was the first randomized controlled study that investigated the relationship between BP lowering and intraparenchymal hemorrhage in 324 participants. In 2019, the conclusion of the BP‐TARGET trial suggested that an intensive SBP target (100–129 mm Hg) did not reduce the rate of ICH at 24–36 h compared with a conventional SBP target (130–185 mm Hg).^15^ Therefore, an intensive BP target of less than 130 mm Hg is unnecessary to limit the risk of hemorrhage. However, the primary outcome of this trial is an evaluation index for short‐term safety, and there is a lack of adequate evidence for the long‐term functional outcomes. Furthermore, the conclusion's validity is weakened by the poor rate of obtaining the BP target and the insufficient median percentage time inside the assigned BP target range.

**TABLE 1:** Randomized controlled trials of blood pressure targets after endovascular thrombectomy: Characteristics and main results.

Trial name	Year and study state	BP target	Number of participants	Primary outcome
BP‐TARGET	Completed (published in 2021)	SBP < 130 mm Hg (*n* = 158) vs SBP < 185 mmHg (*n* = 160)	324	ICH at 24–36 h: SBP < 130, 42% vs SBP < 185, 43% (aOR = 0·96 [0·60–1·51]; *p* = 0·84)
ENCHANETED 2/MT	Terminated (published in 2022)	SBP <120 mmHg (*n* = 407) vs SBP 140‐180 mmHg (*n* = 409)	816	SBP <120 mmHg group had worse 90 days mRS (OR = 1·37 [1·07–1·76]; *p* = 0.01), more early neurological deterioration (OR = 1·53 [1·18–1·97]; *p* = 0.001), more major disability (OR = 2·07 [1·47–2·93]; *p* < 0.0001) at 90 days; sICH: no significant differences
BEST‐II	Completed (published in 2023)	SBP≤180 mmHg (*n* = 40) vs SBP < 160 mmHg (*n* = 40) vsSBP <140 mmHg (*n* = 40)	120	Follow‐up infarct volume at 36 h ± 12 h: SBP < 140, 32.4 mL vs SBP < 160, 50.7 mL vs SBP ≤180, 46.4 mL Utility‐weighted mRS score at 90 d: SBP < 140, 0.51 vs SBP < 160, 0.47 vs SBP ≤180, 0.58
OPTIMAL	Active, not recruiting (published in 2023)	SBP <140 mmHg (*n* = 155) vs SBP < 180 mmHg (*n* = 147)	306	90d mRS Functional independence: SBP <140, 39.4% vs SBP < 180, 54.4% (aOR = 0.56 [0.33–0.96]; *p* = 0.03). Rates of sICH: SBP <140, 9.0% vs SBP < 180, 8.1% (aOR = 1.10 [0.48–2.53]; *p* = 0.82).
DETECT	Completed (unpublished)	SBP <140 mmHg vs SBP <180 mmHg	30	Mean enrollment rate; Number of participants with treatment location change
CRISIS‐I	Terminated (unpublished)	SBP < 120 mmHg vs SBP <140 mmHg	205	90d mRS
HOPE	Recruiting	Control Group: with rt‐PA: SBP < 180/110 mmHg without rt‐PA: SBP < 200/120 mmHg Vs Intervention Group: TICI 2b: SBP 140–160 mmHg TICI 2c‐3: SBP < 140 mmHg.	814	90d mRS
CLEVER	Recruiting	Control Group: Target BP of 90‐120 mmHg Intervention Group: Target BP of 90–160 mmHg	80	Time to target BP; Incidence of any hemorrhagic conversion at 24 h

Abbreviations: ICH, intracranial hemorrhage; mRS, modified Rankin Scale; rt‐PA, alteplase; SBP, systolic blood pressure; sICH, symptomatic intracranial hemorrhage; TICI, thrombolysis in cerebral infarction.

The ENCHANTED 2/MT clinical trial, with 821 participants, randomly assigned patients to either a more intensive BP lowering target (SBP <120 mm Hg) or a less intensive BP lowering target (SBP 140–180 mm Hg). This large‐scale clinical trial was terminated early because it concluded that a more intensive BP lowering target than 120 mm Hg resulted in significantly greater neurological deterioration within 7 days and worse functional outcomes than less intensive SBP control (140–180 mm Hg).^16^ Similar to the BP‐TARGET trial, they found no differences in mortality, serious adverse events, or symptomatic ICH between groups. SBP <120 mmHg was first regarded as the bottom line of safety for BP management after mechanical thrombectomy.

Simultaneous reporting of the OPTIMAL‐BP and BEST II trials' results provided substantial support for BP treatment following EVT.^17^, ^18^ However, the simultaneous publication of these two articles shifted the balance from positive to negative much more. In a prospective multisite observational cohort analysis, Mistry et al. determined that a peak post‐EVT SBP of 158 mm Hg was the threshold to distinguish between good and bad functional outcomes.^14^ BESTII is a futility‐design, randomized, phase 2 clinical trial that included 120 patients with successful EVT for acute ischemic stroke (AIS) to determine the futility of lower SBP targets after endovascular therapy (<140 mm Hg or 160 mm Hg) compared to higher target (180 mm Hg). Two lower BP target groups suggested worse functional outcomes than the higher one, but there is no difference in the rates of any ICH between them. According to statistical prediction, when the sample size is increased to 1500, the probability of achieving a future superiority in moderate SBP lowering target is 25%, which is close to the prespecified utility threshold. As for less than 160 mm Hg, the probability was even lower. Worse still, the probability of success of a future superiority trial did not show an obvious increase by expanding sample size, suggesting this predicted benefit is probably the result of data variability rather than the actual benefit. OPTIMAL is a randomized, phase 3 trial conducted by Ham et al. in South Korea, which was designed to enroll 668 patients with AIS who achieved successful reperfusion after EVT, but was terminated in advance after enrolling 306 patients due to safety concerns brought up by the ENCHANTED2/MT results. Patients were randomized to intensive (SBP target <140 mm Hg) or conventional (SBP target 140–180 mm Hg) management group. This trial showed that the intensive BP management group had poorer functional results than the conventional BP management group and malignant cerebral edema happened more frequently in the intensive BP management group, but symptomatic ICH and morality were similar between groups.

Clinical trials have tested and evaluated either rigorous or moderate BP targets ranging from less than 160 mmHg to 140 mmHg, then 135 mmHg, and finally less than 120 mmHg. In summary, two consensuses can be drawn from them. One of the conclusions is that an intensive BP lowering strategy is not recommended because the findings of the recently completed clinical trials failed to demonstrate the beneficial impact of intensive BP control after successful EVT on the functional outcome. The other is that, when BP was lowered below 180 mm Hg, the incidence of ICH was low and the risk was not significantly different across BP target groups.

## 3 INTERPRETATION OF INCONSISTENT RESULT

The observational studies have shown that the lower SBP after successful EVT may be associated with better functional outcomes and less ICH. Unfortunately, the participants in the RCTs do not appear to benefit from SBP lowering in improving functional outcome and decreasing the risk of ICH as cohort studies. Except for the design and execution flow, there are various possible explanations for the disparities in the result of intensive BP management between observational studies and randomized controlled studies.

Firstly, the relationship between BP and cerebral blood flow (CBF) is complex and non‐monotonic. In normal physiological conditions, autoregulation of CBF is a protective and universal mechanism that maintains a relatively stable blood flow to the brain despite fluctuations in BP by regulating cerebrovascular resistance. The regulation curve is sigmoid with a plateau across a wide range of BP levels (50–150 mmHg in mean arterial pressure).^19^, ^20^ This kind of regulation mechanism, defined as static cerebral autoregulation (CA), occurs mainly when BP or intracranial pressure changes slowly. As BP varies rapidly (minutes or seconds), the CBF becomes incrementally unstable and shows significant fluctuations. Dynamic CA plays an important role in transient and instant changes in BP.

**FIGURE 1:**
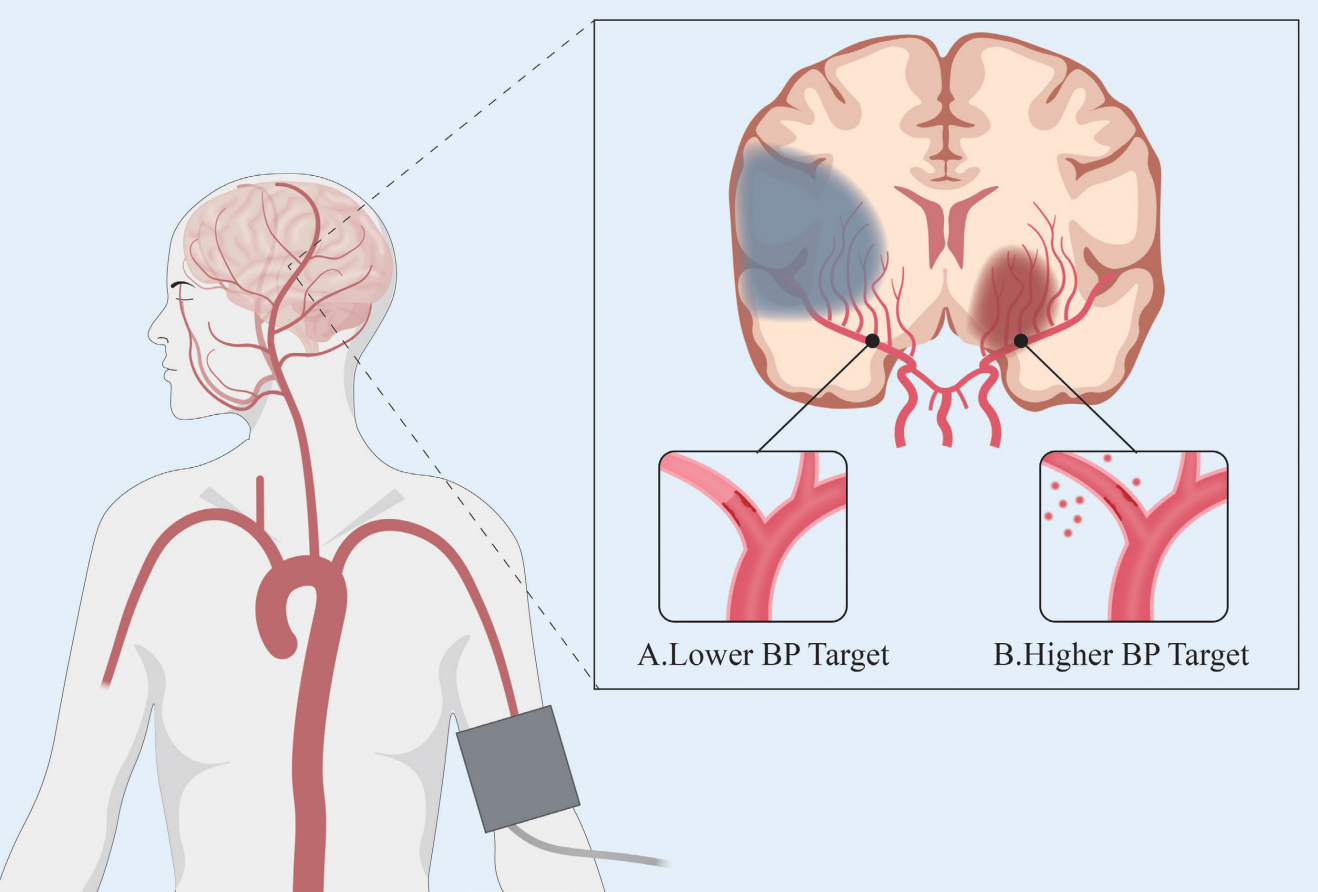
The potential risk of lower or higher blood pressure target after endovascular thrombectomy. In pathophysiology, a lower blood pressure target may impair cerebral perfusion, leading to ischemia and increased infarction (A). However, a higher blood pressure target may cause cerebral edema and cerebral hemorrhage (B). **BP** , blood pressure.

In the setting of LVO, both static and dynamic CA are transiently impaired because of ischemia and hypoxia, which makes the CBF more susceptible to BP fluctuations.^20^, ^21^, ^22^ Due to a variety of factors, such as elevated serum catecholamine levels and impaired baroreceptor sensitivity, more than two‐thirds of patients show an acute elevation of BP, causing an increase in CBF.^23^, ^24^ Furthermore, ischemia and reperfusion injury and mechanical damage to the vessel wall during EVT may make the reperfused blood vessels more vulnerable, leading to blood‐brain barrier disruption, cerebral edema, and subsequent cerebral hemorrhage.^25^, ^26^, ^27^ Thus, the feasibility of lowering BP is supported in theory. In contrast, CBF becomes more dependent on BP when cerebral autoregulation is dysfunctional.^22^, ^28^, ^29^ At the same time, microcirculation disturbance after AIS also reduces brain perfusion. As a consequence, lowering BP in these conditions increases the risk of ischemic damage and even hypoperfusion^30^ (Figure 1).

However, BP is the primary factor that influences cerebral perfusion but not the only one. In patients with successful recanalization after EVT, there are intricate interactions between BP, collateral status, infarction volume, reperfusion, and clinical prognosis. Large acute ischemic stroke volume or more severe ischemia, and poor collateral status (CS) and inadequate recanalization are associated with worse outcomes and accompanied with higher BP usually.^31^, ^32^, ^33^, ^34^ In a post‐hoc analysis of SWIFT trial, David et al. found that better CS was associated with lower baseline BP, smaller baseline infarcts, greater likelihood of successful revascularization, and good clinical outcomes.^35^ In the above situations, a compensatory evaluation in BP is regulated to supplement the lacking CBF. This reverse causality may confound the association between poor outcomes and elevated BP, which could be the cause of the correlation between high BP and poor prognosis that observational studies revealed. Elevated BP may be a normal reaction to this dynamic relationship, and intensive BP lowering may disrupt the body's normal regulation, resulting in negative effects. This hypothesis could also be reflected in the overall trend of BP changes, which elevates in the acute period and then gradually drops after recanalization.

Another possible explanation with a glimmer of hope is that the BP window could be narrower and other BP ranges could be tried. But in our opinion, this endeavor may be in vain. The BP intervals have already been close in the recent RCTs. Nonetheless, it is still unknown what the best level of BP control for optimum outcomes after EVT in AIS is. Furthermore, it is difficult to maintain the BP within a specific range. In these RCTs, the achieved mean SBPs in all groups are lower than 140 mmHg, and the sustaining duration in the target SBP is too short, which may be brought by spontaneous BP lowering.^15^, ^16^, ^17^, ^18^ The discrepancy between prespecified BP targets and achieved BP levels may reduce effectiveness. In addition, the differences between all target groups were less than 10 mmHg and the crossover of actual BP between groups indicates that maintaining the BP in the fixed range is difficult, much less restricting it to a narrower range.

## 4 FUTURE DIRECTION OF BP MANAGEMENT AFTER EVT

### 4.1 Individualized BP management

In addition to the two explanations above, a vital issue should not be ignored. It is not desirable to fully simplify the complex contradictions between BP management and prognosis once and for all by finding the ideal BP that is appropriate for everyone with successful EVT. Instead of oversimplifying the complicated physiologic situation following EVT, such as targeting a single ideal BP value or just stratifying by reperfusion status, individualized therapy is a better alternative. Specifically, we should select patients who are most likely to benefit from BP lowering before practicing different intensities of antihypertensive treatment. Moreover, target SBP and DBP levels need to be individualized, combining collateral status, infarction volume, reperfusion, chronic hypertension history, etc.

Patients' selection has already been executed in recent studies. In the inclusion criteria of OPTIMAL and ENCHANTED 2/MT trial, eligible participants must have elevated SBP (≥140 mmHg) in two or more consecutive readings within a few hours of successful reperfusion.^16^, ^17^ A more stringent definition of sustained elevated SBP level after recanalization was implemented in the ongoing DETECT trial, which screens patients with two consecutive systolic BP readings of higher than 150 mmHg or higher than 140 mmHg if the participant has a history of hypertension. In the uncompleted HOPE trials, SBP target objects are determined by the degree of recanalization after thrombectomy, which may provide additional evidence for intensive BP management following EVT. In addition, describing and exploring the characteristics of BP change in first 24 h after EVT in patients with different functional outcomes could be a future direction to identify the appropriate population. ^36^Petersen et al. found that BP follows specific trajectories after EVT.^36^ They separated five distinct postprocedural SBP trajectories in the first 72 h after EVT and demonstrated that these distinct trajectories have differing associations with functional outcomes. Similarly, Aristeidis et al. identified 4 distinct SBP trajectories in the first 24 h after EVT and found that persistently higher SBP trajectory (mean SBP, 160 mm Hg) is linked to unfavorable outcomes both in the short and long term in comparison with low or moderate SBP trajectories.^37^ Future research could focus on identifying patients with high‐risk trajectories and excluding the patients with high risk of hypotensive episodes.

Several studies have already been applied individualized BP targets in different patients. The ongoing HOPE trial chooses individual BP targets after EVT by the degree of recanalization after thrombectomy, which may provide additional evidence for intensive BP management following EVT. The RCTs of BP lowering during EVT has provided some evidence in applying individualized BP management. Endovascular Thrombectomy in Individualized Blood Pressure Management During Endovascular Stroke Treatment (INDIVIDUAL) trial and Effect of Individualized Versus Standard BP Management During MT for Anterior Ischemic Stroke (DETERMINE) trial (NCT04352296) aim to keep BP at the preinterventional level.^38^, ^39^ In the INDIVIDUAL trial, researchers implement a fixed SBP range of 140 – 180 mm Hg in the standard treatment group and keep the intraprocedural systolic BP at the level of the baseline value ±10 mm Hg in the individualized intervention group. There was no significant difference in the favorable functional outcomes between the two groups. Although the two arms of the study had similar ranges of the mean BP values, there was less statistical power to detect treatment effects in different BP techniques.^38^ In addition, BP control guided by autoregulation may offer an innovative substitute for the traditional method of keeping BP below a set, predefined level, such as near‐infrared spectroscopy‐derived tissue oxygenation and transcranial doppler.^40^, ^41^ It is already proven to be a more effective strategy than static systolic BP thresholds by Petersen et al.'s studies.^41^

### 4.2 Blood pressure variability

Apart from hypertension and hypotension, blood pressure variability (BPV) is another independent factor indicating BP dysregulation, representing the degree of BP fluctuation over time.^42^, ^43^ Impaired cerebral autoregulation after acute LVO results in a progressive change of BP over the first hours, as well as fast, short‐lasting BP fluctuations. The observational studies have shown that parameters describing SBP variability, including SBP standard deviation (SD), SBP reduction (100*[admission SBP‐mean SBP/admission SBP]), SBP range, maximum SBP, and coefficient variation (CV) of SBP, have been shown to be associated with poor functional outcomes in an observational study.^7^, ^44^

Nowadays, the grouping of current RCTs is based on SBP, and the results have not supported intensive BP lowering. Therefore, we need to consider exploring BPV as a novel target in future RCTs, apart from traditional SBP‐based targets. Research on the selection and management of patients with high BPV is still relatively scarce. The development of BPV prediction models and rapid assessment through spectral analysis are important directions for future exploration, and autoregulation‐guided BP control also plays a significant role in reducing BPV.

Reducing variability could avoid hyper‐ or hypoperfusion and provide stable cerebral perfusion. Therefore, we should limit the use of potent, short‐acting antihypertensive drugs and adhere to previous medication treatments as much as possible to avoid increasing iatrogenic BPV and hypotension. Studies have shown that calcium antagonists and non‐benzodiazepine diuretics have been proven to reduce BPV, while β‐blockers increase BPV.^45^, ^46^ There are differences in the antihypertensive drugs used in these four RCTs due to differences in physician practices and clinical guidelines around the world. Currently, there is no clear consensus on the best antihypertensive drug for EVT. The ongoing CLEVER study is aimed at assessing the safety and efficacy of intensive BP control only using Clevidipine, a kind of calcium antagonist, in AIS patients undergoing EVT within 24 h of symptoms onset. Research comparing the treatment effects of different antihypertensive drugs in patients after EVT is necessary.

### 4.3 Multi‐stage BP management

Apart from BP after EVT, several studies have shown that BP parameters before and during EVT are also associated with poor outcomes.^47^, ^48^ BP management before, during, and early stage after EVT should be regarded as continuous rather than separate three stages. It is worth noting that current RCTs about BP management in patients undergoing successful EVT have only focused on one of these stages. Future BP intervention research should focus on the integrated management of the above three phases.

## 5 CONCLUSION

Preoperative BP, intraoperative BP, and early postoperative BP management are the three unalienable stages of perioperative BP management for endovascular therapy. BP management during the three phases has a combined effect on the prognosis. Nowadays, some studies only focus on one of these stages, and longer‐duration BP intervention experiments should be investigated in the future. Stratification using more relevant elements is still necessary for validation, even though the current findings do not provide evidence for an advantage of the intensive BP lowering strategy compared with the conventional strategy. In future research, it is of great significance to identify patients who require BP lowering as early as possible and implement individual BP targets for patients in different states.

## FUNDING INFORMATION

This research was supported by the National Natural Science Foundation of China (82,071,468,82,271,507), Beijing Physician Scientist Training Project (BJPSTP‐2024‐04), and Beijing Science Foundation for Distinguished Young Scholars (JQ24041).

## CONFLICT OF INTEREST STATEMENT

There are no conflicts of interest to declare.

## Data Availability

Data sharing is not applicable to this article as no new data were created or analyzed in this study.
